# Leveraging Digital Platforms: An Eight-Webinar Teaching Series to Enhance Organised Learning in Orthopaedic Surgery

**DOI:** 10.7759/cureus.73657

**Published:** 2024-11-14

**Authors:** Omar Desouky

**Affiliations:** 1 Royal Preston Hospital, Lancashire Teaching Hospitals NHS Foundation Trust, Preston, GBR

**Keywords:** customized medical education, online medical education, online teaching, post grad medical education, webinar series

## Abstract

Introduction

The increasing use of technology and the adoption of online learning platforms have provided more options for delivering medical education. We sought to evaluate the effectiveness of a tailored eight-session orthopaedic webinar series designed to enhance knowledge and confidence among medical students, postgraduate trainees, and healthcare providers involved in musculoskeletal care.

Methods

A total of 427 participants from 43 countries attended the webinar series. Quantitative feedback was collected through questionnaires assessing previous orthopaedic teaching sufficiency, preparedness before encountering orthopaedic cases, confidence levels before and after sessions, engagement, relevance to learning needs, and interest in the format. Qualitative feedback was also gathered to identify strengths and areas for improvement.

Results

Participants reported low sufficiency in prior orthopaedic teaching (average score: 2.86) and felt underprepared before attending the sessions (average score: 2.48). Confidence levels significantly increased after the sessions, rising from an average of 2.61 before to 3.70 after. High engagement (average score: 4.23) and interest in the format (average score: 4.45) were noted. Qualitative feedback highlighted the value of case-based learning, interactive elements, and practical insights into patient management. Areas for improvement included addressing technical issues and incorporating more interactive features like quizzes or polls.

Conclusion

The webinar series effectively improved participants' confidence and understanding of key orthopaedic concepts. Overall, there were high engagement scores and interest in the format. This highlights that structured, tailored, and interactive online webinars could help supplement knowledge, especially in areas where the participants had decreased exposure during their training.

## Introduction

The use of digital technology has significantly transformed medical education, with the use of online learning platforms accelerating rapidly, particularly in response to the constraints posed by the COVID-19 pandemic [1,2]. This global health crisis necessitated an abrupt shift from traditional face-to-face instruction to virtual learning environments, fundamentally altering the modalities of medical education [1,3]. 

Online education, once considered supplementary, has now become an essential component of medical training [2,4]. It offers unique advantages over traditional in-person learning, including broader accessibility, scalability, and the flexibility to engage remotely [5,6]. These benefits enable diverse, global audiences to participate in learning without geographic limitations, addressing an essential demand in modern healthcare training [4,7]. Remote learning platforms have allowed medical education to continue uninterrupted, providing students with access to educational resources regardless of their location [4,8].

Well-designed online platforms can effectively support or enhance knowledge retention and skills acquisition [9,10]. Interactive, case-based learning and virtual simulations cultivate critical thinking and decision-making skills essential for clinical practice [11,12]. Virtual simulations and interactive online modules could effectively replace certain aspects of clinical training without compromising educational outcomes [11]. Pei and Wu conducted a systematic review and meta-analysis, concluding that online learning is as effective as traditional offline learning in undergraduate medical education [5].

Platforms such as Zoom and Google Classroom have proven effective for group discussions and case-based scenarios, supporting real-time collaboration among geographically dispersed participants [12]. These platforms can facilitate synchronous learning experiences, allowing for immediate feedback and interaction, which are crucial components of effective learning [12]. O'Doherty et al. also highlighted the importance of interactive elements in online learning to enhance engagement and knowledge retention [7].

Despite these advantages, online education in medicine faces challenges. Technical barriers, including unstable internet connections and limited access to necessary technology, can hinder interaction and reduce learning efficacy [13,14]. Students from low- and middle-income countries could face significant obstacles due to technological limitations, impacting their learning experience [13]. Additionally, the absence of face-to-face interactions may affect relationship-building and peer support, essential elements in professional identity formation among medical students [15]. The lack of direct interaction can also lead to feelings of isolation and decreased motivation among learners [14].

While online platforms excel in accessibility, they may lack the social connectivity inherent to in-person learning environments, a factor crucial for engagement and emotional support [16,17]. Torda and Shulruf compared online and face-to-face small-group teaching, finding that while content delivery could be equivalent, the nuances of in-person interactions are difficult to replicate online [16]. Khalil et al. explored medical students' perspectives on the sudden transition to synchronised online learning and highlighted the challenges in maintaining engagement [15].

Nevertheless, online education, particularly through tailored, topic-specific webinars, shows strong potential for addressing specialty-specific knowledge gaps [17]. Customisable webinar series enables content tailored to varied experience levels and professional backgrounds, supporting the development of a competent and well-rounded healthcare workforce [9,18]. Webinars could effectively supplement medical education during times when traditional learning is disrupted, providing flexibility and focused content delivery [17]. A meta-analysis demonstrated that internet-based learning in the health professions is associated with positive educational outcomes, supporting the effectiveness of online educational interventions [18].

The lack of orthopaedic and musculoskeletal education in undergraduate medical curricula is well documented [19-22]. Research indicates that many medical graduates lack basic musculoskeletal knowledge due to minimal clinical exposure and limited dedicated lecture hours during training [19-20]. This can affect their confidence and competency in clinical practice, as highlighted in studies across the US, UK, and Canada [19-21]. Al-Nammari et al. found that junior doctors in the UK demonstrated inadequate musculoskeletal knowledge after foundation training [20]. Similarly, Pinney and Regan noted that Canadian medical curricula did not sufficiently reflect community needs regarding musculoskeletal problems [21].

Addressing this need for enhanced orthopaedic knowledge and confidence, we developed a tailored eight-session webinar series. This series aimed to provide a targeted and interactive learning experience to bridge the educational gap in musculoskeletal medicine. By offering an exploration of essential orthopaedic concepts tailored for medical students, postgraduate trainees, and other healthcare providers involved in musculoskeletal care, the series sought to improve competency and confidence in this crucial area of medicine.

## Materials and methods

Structure of the webinar series

The webinar series was organised over eight sessions, each addressing a different topic. Topics included orthopaedic emergencies, acute joint swelling, femoral fractures and management, paediatric conditions and safeguarding, perioperative anticoagulation, spinal emergencies, ankle fractures, and wrist and forearm fractures.

The sessions utilised a lecture-based format, complemented by clinical case discussions, Q&A sessions, and interactive polls or quizzes when appropriate. Visual aids, including X-rays and anatomical illustrations, were integral to demonstrating real-life scenarios and enhancing participant engagement.

Participants

Participants represented a wide range of healthcare roles working across a range of specialties, including emergency medicine, urgent care, community and primary care, and trainees in secondary care with exposure to orthopaedic cases. The roles included foundation year doctors, core trainees, registrars, general practitioners, nurses and advanced practitioners, physiotherapists, paramedics, and radiologists. Their backgrounds varied from minimal orthopaedic experience to advanced practice, allowing a rich diversity of perspectives and engagement levels.

Data collection

The data on participant experience was collected via post-session surveys, which included both quantitative and qualitative measures. Demographic information on participants' professions, grades, and country of origin was also gathered. Participants were asked to provide feedback on seven questions designed to assess their perceptions of the orthopaedic webinar series. These questions were structured using a Likert scale model, allowing participants to rate their responses from one to five stars. The questions addressed areas such as the adequacy of orthopaedic teaching received, preparedness before starting on orthopaedic cases, confidence in the topic before and after the session, engagement with the teaching, relevance of the content, and interest in the format. Average scores were calculated based on these ratings to gauge participants’ satisfaction and perceived impact of the sessions on their knowledge and confidence in orthopaedic care.

Participants were also asked open-ended questions to gather further insights. They provided responses on what aspects of the session went well, areas that could be improved, and suggestions for future topics they would like covered. This feedback allowed us to dynamically adjust and refine the webinar series in response to participant needs and preferences, ensuring the content remained relevant and tailored to their evolving interests and clinical requirements.

## Results

A total of 427 participants attended the eight-session orthopaedic webinar series, with participation rates ranging from 18 to 117 per session. There was a diverse geographical spread among participants, with individuals joining the webinar series from a wide range of countries. In total, 296 participants provided their location. The largest cohort was from the UK, comprising 37.2% of total attendees, while other significant representations included Romania (7.1%), Tunisia (4.7%), Malaysia (4.4%), and Portugal (4.4%). Overall, participants came from 43 different countries across all sessions. 

Quantitative feedback

Participant feedback was collected via a questionnaire, and the data was analysed for each session. Weighted averages were calculated to account for the variation in participant numbers across sessions. Questions and their respective average scores are presented below (Table 1).

**Table 1:** Questions and respective average score

Question	Average score
Is the amount of orthopaedic teaching you previously received sufficient?	2.86
How prepared did you feel before you started seeing orthopaedic cases?	2.48
How confident did you feel about the topic before the session?	2.61
How confident do you feel about the topic after the session?	3.70
How engaging did you find the session?	4.23
How relevant was the teaching to your learning needs?	3.95
How interesting did you find the format?	4.45

Participants provided feedback on the orthopaedic teaching sessions through a series of evaluative questions. When asked about the sufficiency of previous orthopaedic teaching they had received, respondents reported an average rating of 2.86, suggesting a perceived need for additional instruction in this area.

In terms of their initial preparation before encountering orthopaedic cases, participants rated their preparedness with an average score of 2.48, indicating a general feeling of limited readiness. Confidence in the subject matter prior to the session averaged at 2.61, with confidence scores for specific sessions ranging from 2.5 (notably for sessions on femoral fractures, ankle fracture management, paediatric presentations, and spinal emergencies) to 3.0 (seen in sessions on acute joint swelling, orthopaedic emergencies, and wrist fractures).

After completing the sessions, participants’ confidence notably increased, with an average post-session confidence score of 3.70. This improvement was reflected in individual session scores, ranging from 3.5 for femoral fractures, ankle fracture management, and paediatric presentations up to 4.0 for sessions on acute joint swelling, orthopaedic emergencies, spinal emergencies, and wrist fractures.

When asked to evaluate the engagement level of the sessions, participants provided an average rating of 4.23. The lowest engagement scores, at 4.0, were given to sessions on femoral fractures, acute joint swelling, orthopaedic emergencies, and spinal emergencies, while the highest scores, at 4.5, were recorded for sessions on ankle fracture management, paediatric presentations, perioperative anticoagulation, and wrist fractures.

Participants also rated the relevance of the teaching sessions to their learning needs, with an average score of 3.95. Specific sessions such as femoral fractures, ankle fracture management, orthopaedic emergencies, and wrist fractures scored 4.0 in relevance, while acute joint swelling and spinal emergencies sessions reached a higher relevance score of 4.5. When assessing the interest generated by the format of the sessions, respondents gave an average rating of 4.45. While sessions on orthopaedic emergencies and perioperative anticoagulation scored 4.0, most other sessions achieved the maximum interest score of 4.5.

Qualitative feedback

The analysis of qualitative feedback revealed several recurring themes among participants. Attendees highlighted multiple positive aspects of the sessions, including the value of case-based learning. The use of real-life case scenarios was particularly appreciated for providing practical insights into patient management. Additionally, participants praised the interactive elements of the sessions, noting that the inclusion of Q&A opportunities and chat-based interactions significantly enhanced their learning experience. Visual aids and clear, step-by-step explanations were also highly valued, especially for complex topics such as spinal emergencies.

However, attendees also identified areas for improvement. Technical issues, such as occasional audio and connectivity problems, were reported by some participants, as these disruptions detracted from the overall learning experience. Furthermore, there was a frequent call for additional clinical cases; participants expressed a desire for more case discussions and practical examples to further consolidate their understanding. Some attendees also suggested that more interactive features, like quizzes or polls, could be incorporated to increase engagement, especially for topics that were conceptually challenging.

## Discussion

The findings from this eight-session orthopaedic webinar series underscore the value of structured, interactive online learning for healthcare professionals worldwide. With 427 participants from 43 different countries, the series not only demonstrated extensive reach but also effectively improved participants' confidence and understanding of key orthopaedic concepts. These outcomes align with existing literature emphasising the potential of online education to enhance knowledge and skills in medical training [5,11].

Participants reported a perceived insufficiency in their prior orthopaedic education, with an average rating of 2.86 out of 5 regarding the adequacy of previous teaching. This sentiment reflects a well-documented gap in musculoskeletal education within undergraduate medical curricula [19-22]. Previous findings suggested medical graduates lack basic musculoskeletal knowledge due to limited clinical exposure and insufficient dedicated lecture hours [19,20]. Our findings reinforce the importance of supplementary educational initiatives to address these deficiencies.

The significant increase in participants' confidence from an average of 2.61 before the sessions to 3.70 afterward indicates that the webinar series was effective in enhancing self-assessed competency. This improvement is consistent with previous findings demonstrating the efficacy of online learning in medical education [5,18]. Online learning could be as effective as traditional methods in undergraduate medical education, particularly when interactive and well-structured [5]. In general, it could lead to positive educational outcomes across health professions [18].

High engagement scores (average of 4.23) and interest in the format (average of 4.45) suggest that interactive elements such as quizzes, Q&A sessions, and chat interactions contribute significantly to learner engagement. The importance of synchronous learning experiences and immediate feedback in virtual teaching environments has been previously highlighted [12]. The positive reception of these interactive components aligns with adult learning theories, which stress active participation and practical relevance as keys to effective learning [9,10].

The diverse geographical representation among participants highlights the capability of online platforms to overcome traditional barriers to medical education [2,4]. With attendees from countries including the UK, Romania, Tunisia, Malaysia, and Portugal, the webinar series facilitated a global exchange of knowledge. This accessibility aligns with previous observations that remote learning platforms enable continued education irrespective of geographical constraints [4]. Such global reach could be valuable in standardising medical knowledge and practices across different healthcare systems.

Despite the overall success, participants identified technical issues, such as audio and connectivity problems, as areas needing improvement. These challenges are consistent with known barriers to online education [7,13,14]. Technical difficulties can hinder interaction and reduce learning efficacy [7]. Additionally, participants expressed a desire for more interactive features like more quizzes or polls to further enhance engagement, especially for complex topics. Incorporating these elements could address the need for formative assessment and immediate feedback, which are critical for effective learning [9].

The absence of face-to-face interaction was also highlighted as a limitation, potentially impacting relationship-building and professional identity formation [15,17]. The lack of direct interaction might lead to feelings of isolation among learners [14]. Strategies to foster community and connection in virtual environments should be considered to mitigate these effects.

The positive outcomes of this webinar series suggest that online, interactive learning can effectively supplement traditional medical education, particularly in areas where curricular gaps exist [11-17]. By enhancing confidence and perceived competence in orthopaedics, such initiatives can contribute to better preparedness among medical professionals. This is crucial given the identified deficiencies in musculoskeletal education and the importance of orthopaedic knowledge in clinical practice [19-22]. Moreover, the scalability and accessibility of online platforms present an opportunity to widen the reach of medical education globally [2,6]. 

Limitations

There are several limitations that should be kept in mind when interpreting the results. The self-reported nature of the feedback may introduce bias, as participants who chose to provide feedback might have had particularly positive or negative experiences. Additionally, the lack of a control group limits the ability to attribute improvements solely to the webinar series. Objective assessments of knowledge and skills acquisition, such as pre- and post-tests or long-term retention evaluations, were not conducted. Technical issues such as audio and connectivity problems were reported qualitatively but not systematically measured or addressed. These issues could have affected the learning experience for some participants and may have led to disparities in engagement and knowledge acquisition. Future studies could incorporate objective measures of knowledge acquisition and retention to provide more robust evidence of effectiveness.

## Conclusions

This eight-session webinar series successfully addressed a gap in orthopaedic education by providing an interactive, comprehensive learning experience for a diverse group of healthcare professionals. The increased confidence and positive feedback from participants underscore the effectiveness of structured, tailored, interactive online webinar sessions that are easy to attend. These findings highlight the importance of targeted educational initiatives, offering practical, interactive learning opportunities that benefit a broad spectrum of healthcare roles.
